# *N*′-[1-(2-Hydroxy­phen­yl)ethyl­idene]-2-nitro­benzohydrazide methanol solvate

**DOI:** 10.1107/S160053680900508X

**Published:** 2009-02-25

**Authors:** Ge-Jiang Xiao, Chao Wei

**Affiliations:** a School of Chemistry and Biological Engineering, Changsha University of Science and Technology, Changsha, Hunan 410004, People’s Republic of China

## Abstract

In the title compound, C_15_H_13_N_3_O_4_·CH_3_OH, the dihedral angle between the two substituted benzene rings is 66.7 (2)°. An intra­molecular O—H⋯N hydrogen bond is observed in the Schiff base mol­ecule. In the crystal structure, the Schiff base and solvent mol­ecules are linked into chains running along the *a* axis by inter­molecular O—H⋯O and N—H⋯O hydrogen bonds.

## Related literature

For the biological properties of hydrazone compounds, see: Bedia *et al.* (2006 ▶). For complexes of hydrazone compounds, see: Iskander *et al.* (2001 ▶); Aggarwal *et al.* (1981 ▶); Aruffo *et al.* (1982 ▶). For related structures, see: Fun *et al.* (2008*a* ▶,*b* ▶); Butcher *et al.* (2007 ▶); Zhi & Yang (2007 ▶); Mohd Lair *et al.* (2009*a* ▶,*b* ▶); Yehye *et al.* (2008 ▶). For bond-length data, see: Allen *et al.* (1987 ▶).
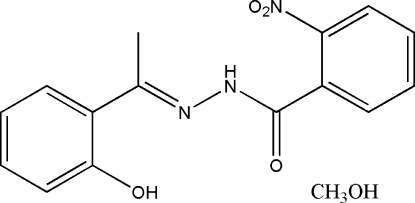


## Experimental

### 

#### Crystal data

C_15_H_13_N_3_O_4_·CH_4_O
*M* *_r_* = 331.33
Triclinic, 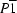

*a* = 7.124 (2) Å
*b* = 8.066 (2) Å
*c* = 15.764 (3) Å
α = 101.950 (2)°
β = 92.972 (2)°
γ = 114.889 (2)°
*V* = 794.0 (3) Å^3^
*Z* = 2
Mo *K*α radiation
μ = 0.11 mm^−1^
*T* = 298 K
0.23 × 0.23 × 0.22 mm

#### Data collection

Bruker SMART 1000 CCD area-detector diffractometer
Absorption correction: multi-scan (*SADABS*; Bruker, 2001 ▶) *T*_min_ = 0.976, *T*_max_ = 0.977
4659 measured reflections
3371 independent reflections
2660 reflections with *I* > 2σ(*I*)
*R*_int_ = 0.014

#### Refinement

*R*[*F* ^2^ > 2σ(*F* ^2^)] = 0.043
*wR*(*F* ^2^) = 0.124
*S* = 1.05
3371 reflections
226 parameters
1 restraint
H atoms treated by a mixture of independent and constrained refinement
Δρ_max_ = 0.24 e Å^−3^
Δρ_min_ = −0.23 e Å^−3^

### 

Data collection: *SMART* (Bruker, 2007 ▶); cell refinement: *SAINT* (Bruker, 2007 ▶); data reduction: *SAINT*; program(s) used to solve structure: *SHELXTL* (Sheldrick, 2008 ▶); program(s) used to refine structure: *SHELXTL*; molecular graphics: *SHELXTL*; software used to prepare material for publication: *SHELXTL*.

## Supplementary Material

Crystal structure: contains datablocks global, I. DOI: 10.1107/S160053680900508X/ci2770sup1.cif

Structure factors: contains datablocks I. DOI: 10.1107/S160053680900508X/ci2770Isup2.hkl

Additional supplementary materials:  crystallographic information; 3D view; checkCIF report

## Figures and Tables

**Table 1:** Hydrogen-bond geometry (Å, °)

*D*—H⋯*A*	*D*—H	H⋯*A*	*D*⋯*A*	*D*—H⋯*A*
N1—H1⋯O5^i^	0.893 (9)	2.08 (1)	2.9563 (17)	165 (2)
O5—H5⋯O1	0.82	1.94	2.7451 (16)	168
O4—H4⋯N2	0.82	1.85	2.5612 (17)	144

Symmetry code: (i) 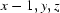.

**Fig. 1.**
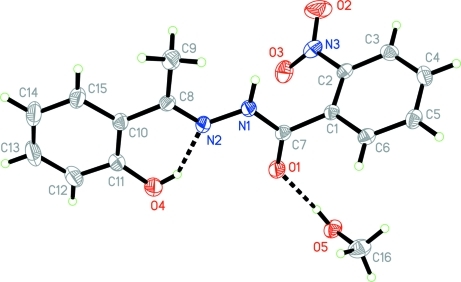
The molecular structure of the title compound, with 30% probability displacement ellipsoids. Hydrogen bonds are shown as dashed lines.

**Fig. 2.**
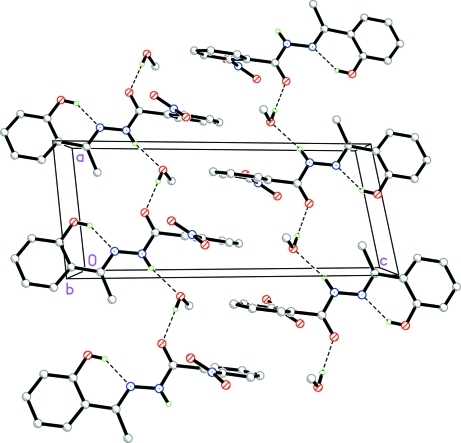
Hydrogen-bonded (dashed lines) chains in the title compound, viewed along the b axis. H atoms not involved in the interactions have been omitted for clarity.

## supplementary crystallographic information

### Comment

Hydrazone compounds have been demonstrated to possess biological properties, such as antimicrobial, antitubercular, anticancer and antitumor (Bedia *et al.*, 2006). Moreover, these compounds are good ligands in the coordination chemistry (Iskander *et al.*, 2001; Aggarwal *et al.*, 1981; Aruffo *et al.*, 1982). Recently, a large number of hydrazone compounds have been reported (Fun *et al.*, 2008*b*; Butcher *et al.*, 2007; Zhi & Yang, 2007). In this paper, a new hydrazone compound (Fig. 1), derived from 1-(2-hydroxyphenyl)ethanone and 2-nitrobenzohydrazide is reported.

The asymmetric unit of the title compound contains a Schiff base molecule and a methanol molecule of crystallization. The dihedral angle between the two substituted benzene rings is 66.7 (2)°, indicating that the Schiff base molecule is twisted. The dihedral angle between the C1-C6 and O2/O3/N3/C2 planes is 26.0 (1)°. All bond lengths in the compound are typical (Allen *et al.*, 1987) and comparable to those observed in similar hydrazone compounds (Fun *et al.*, 2008*a*; Mohd Lair *et al.*, 2009*a*,b; Yehye *et al.*, 2008). An intramolecular O—H···N hydrogen bond is observed in the Schiff base molecule.

In the crystal structure, the Schiff base and methanol molecules are linked through O–H···O and N–H···O hydrogen bonds (Table 1), forming chains running along the *a* axis (Fig. 2).

### Experimental

1-(2-Hydroxyphenyl)ethanone (1.0 mmol, 136.2 mg) and 2-nitrobenzohydrazide (1.0 mmol, 197.2 mg) were stirred at room temperature for 3 h. The filtrate was kept in air for a few days to obtain colourless block-shaped crystals of the title compound.

### Refinement

Atom H1 attached to N1 was located in a difference Fourier map and refined isotropically, with the N–H distance restrained to 0.90 (1) Å. C- and O-bound H atoms were positioned geometrically and refined using a riding model with d(C–H) = 0.93–0.96 Å, d(O–H) = 0.82 Å and *U*_iso_ = 1.2*U*_eq_(C) and 1.5*U*_eq_(O and C_methyl_).

### Crystal data

**Table:**

C_15_H_13_N_3_O_4_·CH_4_O	*Z* = 2
*M**_r_* = 331.33	*F*(000) = 348
Triclinic, *P*1	*D*_x_ = 1.386 Mg m^−^^3^
Hall symbol: -P 1	Mo *K*α radiation, λ = 0.71073 Å
*a* = 7.124 (2) Å	Cell parameters from 1968 reflections
*b* = 8.066 (2) Å	θ = 2.6–28.5°
*c* = 15.764 (3) Å	µ = 0.11 mm^−^^1^
α = 101.950 (2)°	*T* = 298 K
β = 92.972 (2)°	Block, colourless
γ = 114.889 (2)°	0.23 × 0.23 × 0.22 mm
*V* = 794.0 (3) Å^3^	

### Data collection

**Table:**

Bruker SMART 1000 CCD area-detector diffractometer	3371 independent reflections
Radiation source: fine-focus sealed tube	2660 reflections with *I* > 2σ(*I*)
graphite	*R*_int_ = 0.014
ω scans	θ_max_ = 27.0°, θ_min_ = 2.7°
Absorption correction: multi-scan (*SADABS*; Bruker, 2001)	*h* = −8→9
*T*_min_ = 0.976, *T*_max_ = 0.977	*k* = −10→10
4659 measured reflections	*l* = −19→20

### Refinement

**Table:**

Refinement on *F*^2^	Secondary atom site location: difference Fourier map
Least-squares matrix: full	Hydrogen site location: inferred from neighbouring sites
*R*[*F*^2^ > 2σ(*F*^2^)] = 0.043	H atoms treated by a mixture of independent and constrained refinement
*wR*(*F*^2^) = 0.124	*w* = 1/[σ^2^(*F*_o_^2^) + (0.0585*P*)^2^ + 0.1607*P*] where *P* = (*F*_o_^2^ + 2*F*_c_^2^)/3
*S* = 1.05	(Δ/σ)_max_ = 0.001
3371 reflections	Δρ_max_ = 0.24 e Å^−^^3^
226 parameters	Δρ_min_ = −0.23 e Å^−^^3^
1 restraint	Extinction correction: *SHELXTL* (Sheldrick, 2008), Fc^*^=kFc[1+0.001xFc^2^λ^3^/sin(2θ)]^-1/4^
Primary atom site location: structure-invariant direct methods	Extinction coefficient: 0.066 (6)

### Special details

**Table:**

Geometry. All e.s.d.'s (except the e.s.d. in the dihedral angle between two l.s. planes) are estimated using the full covariance matrix. The cell e.s.d.'s are taken into account individually in the estimation of e.s.d.'s in distances, angles and torsion angles; correlations between e.s.d.'s in cell parameters are only used when they are defined by crystal symmetry. An approximate (isotropic) treatment of cell e.s.d.'s is used for estimating e.s.d.'s involving l.s. planes.
Refinement. Refinement of *F*^2^ against ALL reflections. The weighted *R*-factor *wR* and goodness of fit *S* are based on *F*^2^, conventional *R*-factors *R* are based on *F*, with *F* set to zero for negative *F*^2^. The threshold expression of *F*^2^ > σ(*F*^2^) is used only for calculating *R*-factors(gt) *etc*. and is not relevant to the choice of reflections for refinement. *R*-factors based on *F*^2^ are statistically about twice as large as those based on *F*, and *R*- factors based on ALL data will be even larger.

### Fractional atomic coordinates and isotropic or equivalent isotropic displacement parameters (Å^2^)

**Table:**

	*x*	*y*	*z*	*U*_iso_*/*U*_eq_	
O1	0.46363 (18)	0.2874 (2)	0.22886 (7)	0.0558 (3)	
O2	0.2215 (3)	0.7197 (2)	0.41556 (13)	0.0902 (5)	
O3	0.4022 (2)	0.65556 (18)	0.32038 (9)	0.0639 (4)	
O4	0.3900 (2)	0.2749 (2)	−0.00437 (8)	0.0597 (4)	
H4	0.3625	0.2787	0.0457	0.089*	
O5	0.81043 (17)	0.27761 (18)	0.30757 (8)	0.0522 (3)	
H5	0.7158	0.2968	0.2867	0.078*	
N1	0.13992 (19)	0.27191 (19)	0.20210 (8)	0.0396 (3)	
N2	0.1602 (2)	0.27247 (19)	0.11548 (8)	0.0403 (3)	
N3	0.2967 (2)	0.61957 (19)	0.37848 (10)	0.0495 (4)	
C1	0.2777 (2)	0.2991 (2)	0.34961 (9)	0.0336 (3)	
C2	0.2633 (2)	0.4482 (2)	0.40702 (9)	0.0363 (3)	
C3	0.2296 (2)	0.4472 (2)	0.49259 (10)	0.0441 (4)	
H3	0.2163	0.5472	0.5288	0.053*	
C4	0.2163 (2)	0.2946 (2)	0.52303 (10)	0.0472 (4)	
H4A	0.1947	0.2917	0.5805	0.057*	
C5	0.2348 (2)	0.1460 (2)	0.46837 (11)	0.0450 (4)	
H5A	0.2286	0.0448	0.4896	0.054*	
C6	0.2625 (2)	0.1475 (2)	0.38232 (10)	0.0386 (3)	
H6	0.2712	0.0453	0.3458	0.046*	
C7	0.3056 (2)	0.2892 (2)	0.25478 (9)	0.0375 (3)	
C8	0.0013 (2)	0.2459 (2)	0.06163 (9)	0.0383 (3)	
C9	−0.2106 (3)	0.2077 (3)	0.08602 (11)	0.0564 (5)	
H9A	−0.2363	0.1373	0.1298	0.085*	
H9B	−0.3155	0.1361	0.0350	0.085*	
H9C	−0.2160	0.3253	0.1089	0.085*	
C10	0.0411 (3)	0.2564 (2)	−0.02863 (9)	0.0412 (4)	
C11	0.2322 (3)	0.2729 (2)	−0.05640 (10)	0.0468 (4)	
C12	0.2639 (4)	0.2882 (3)	−0.14152 (12)	0.0639 (5)	
H12	0.3905	0.3006	−0.1595	0.077*	
C13	0.1107 (4)	0.2853 (3)	−0.19919 (12)	0.0712 (6)	
H13	0.1341	0.2953	−0.2558	0.085*	
C14	−0.0770 (4)	0.2676 (3)	−0.17371 (12)	0.0678 (6)	
H14	−0.1808	0.2648	−0.2131	0.081*	
C15	−0.1110 (3)	0.2540 (3)	−0.08957 (11)	0.0543 (4)	
H15	−0.2382	0.2429	−0.0728	0.065*	
C16	0.7246 (3)	0.1002 (3)	0.32738 (15)	0.0637 (5)	
H16A	0.8322	0.0598	0.3340	0.096*	
H16B	0.6154	0.0097	0.2806	0.096*	
H16C	0.6675	0.1103	0.3810	0.096*	
H1	0.030 (2)	0.277 (3)	0.2252 (11)	0.052 (5)*	

### Atomic displacement parameters (Å^2^)

**Table:**

	*U*^11^	*U*^22^	*U*^33^	*U*^12^	*U*^13^	*U*^23^
O1	0.0432 (7)	0.0981 (10)	0.0467 (6)	0.0446 (7)	0.0145 (5)	0.0297 (6)
O2	0.1112 (14)	0.0669 (9)	0.1256 (14)	0.0645 (10)	0.0392 (11)	0.0317 (9)
O3	0.0749 (9)	0.0548 (7)	0.0645 (8)	0.0235 (7)	0.0108 (7)	0.0313 (6)
O4	0.0544 (8)	0.0912 (9)	0.0469 (7)	0.0439 (7)	0.0126 (6)	0.0183 (7)
O5	0.0372 (6)	0.0664 (7)	0.0588 (7)	0.0265 (6)	0.0036 (5)	0.0209 (6)
N1	0.0333 (7)	0.0574 (8)	0.0337 (6)	0.0243 (6)	0.0058 (5)	0.0135 (6)
N2	0.0391 (7)	0.0543 (7)	0.0329 (6)	0.0252 (6)	0.0042 (5)	0.0123 (5)
N3	0.0477 (8)	0.0415 (7)	0.0602 (9)	0.0216 (6)	−0.0014 (7)	0.0133 (6)
C1	0.0243 (6)	0.0407 (7)	0.0365 (7)	0.0144 (6)	0.0020 (5)	0.0115 (6)
C2	0.0294 (7)	0.0382 (7)	0.0420 (8)	0.0157 (6)	0.0004 (6)	0.0111 (6)
C3	0.0368 (8)	0.0516 (9)	0.0412 (8)	0.0209 (7)	0.0040 (6)	0.0038 (7)
C4	0.0399 (8)	0.0664 (10)	0.0351 (8)	0.0206 (8)	0.0087 (6)	0.0184 (7)
C5	0.0402 (8)	0.0495 (9)	0.0501 (9)	0.0180 (7)	0.0088 (7)	0.0258 (7)
C6	0.0345 (8)	0.0390 (7)	0.0443 (8)	0.0174 (6)	0.0056 (6)	0.0120 (6)
C7	0.0315 (7)	0.0471 (8)	0.0391 (8)	0.0204 (6)	0.0062 (6)	0.0146 (6)
C8	0.0388 (8)	0.0385 (7)	0.0379 (8)	0.0202 (6)	−0.0001 (6)	0.0050 (6)
C9	0.0400 (9)	0.0804 (12)	0.0470 (9)	0.0277 (9)	−0.0005 (7)	0.0123 (9)
C10	0.0492 (9)	0.0390 (7)	0.0340 (7)	0.0215 (7)	−0.0019 (6)	0.0039 (6)
C11	0.0557 (10)	0.0477 (9)	0.0377 (8)	0.0264 (8)	0.0039 (7)	0.0053 (7)
C12	0.0784 (14)	0.0734 (13)	0.0419 (9)	0.0364 (11)	0.0161 (9)	0.0110 (9)
C13	0.1033 (18)	0.0730 (13)	0.0326 (9)	0.0357 (12)	0.0059 (10)	0.0119 (9)
C14	0.0875 (15)	0.0701 (12)	0.0408 (10)	0.0355 (11)	−0.0158 (9)	0.0084 (9)
C15	0.0589 (11)	0.0597 (10)	0.0424 (9)	0.0286 (9)	−0.0080 (7)	0.0077 (8)
C16	0.0495 (11)	0.0652 (12)	0.0875 (14)	0.0311 (9)	0.0180 (10)	0.0274 (10)

### Geometric parameters (Å, °)

**Table:**

O1—C7	1.2218 (18)	C5—H5A	0.93
O2—N3	1.2172 (19)	C6—H6	0.93
O3—N3	1.2192 (19)	C8—C10	1.478 (2)
O4—C11	1.349 (2)	C8—C9	1.497 (2)
O4—H4	0.82	C9—H9A	0.96
O5—C16	1.410 (2)	C9—H9B	0.96
O5—H5	0.82	C9—H9C	0.96
N1—C7	1.3472 (18)	C10—C15	1.401 (2)
N1—N2	1.3812 (17)	C10—C11	1.412 (2)
N1—H1	0.893 (9)	C11—C12	1.394 (2)
N2—C8	1.2916 (19)	C12—C13	1.373 (3)
N3—C2	1.4705 (19)	C12—H12	0.93
C1—C6	1.389 (2)	C13—C14	1.374 (3)
C1—C2	1.392 (2)	C13—H13	0.93
C1—C7	1.5083 (19)	C14—C15	1.379 (3)
C2—C3	1.383 (2)	C14—H14	0.93
C3—C4	1.380 (2)	C15—H15	0.93
C3—H3	0.93	C16—H16A	0.96
C4—C5	1.384 (2)	C16—H16B	0.96
C4—H4A	0.93	C16—H16C	0.96
C5—C6	1.383 (2)		
			
C11—O4—H4	109.5	C10—C8—C9	120.59 (13)
C16—O5—H5	109.5	C8—C9—H9A	109.5
C7—N1—N2	117.46 (12)	C8—C9—H9B	109.5
C7—N1—H1	119.7 (12)	H9A—C9—H9B	109.5
N2—N1—H1	122.2 (12)	C8—C9—H9C	109.5
C8—N2—N1	119.43 (12)	H9A—C9—H9C	109.5
O2—N3—O3	123.74 (15)	H9B—C9—H9C	109.5
O2—N3—C2	118.16 (15)	C15—C10—C11	117.60 (15)
O3—N3—C2	118.08 (14)	C15—C10—C8	120.27 (15)
C6—C1—C2	117.14 (13)	C11—C10—C8	122.11 (14)
C6—C1—C7	117.87 (13)	O4—C11—C12	116.92 (16)
C2—C1—C7	124.98 (13)	O4—C11—C10	123.35 (14)
C3—C2—C1	122.71 (14)	C12—C11—C10	119.73 (16)
C3—C2—N3	117.65 (14)	C13—C12—C11	120.8 (2)
C1—C2—N3	119.55 (13)	C13—C12—H12	119.6
C4—C3—C2	118.57 (15)	C11—C12—H12	119.6
C4—C3—H3	120.7	C12—C13—C14	120.37 (18)
C2—C3—H3	120.7	C12—C13—H13	119.8
C3—C4—C5	120.24 (14)	C14—C13—H13	119.8
C3—C4—H4A	119.9	C13—C14—C15	119.77 (18)
C5—C4—H4A	119.9	C13—C14—H14	120.1
C6—C5—C4	120.21 (14)	C15—C14—H14	120.1
C6—C5—H5A	119.9	C14—C15—C10	121.72 (19)
C4—C5—H5A	119.9	C14—C15—H15	119.1
C5—C6—C1	121.08 (14)	C10—C15—H15	119.1
C5—C6—H6	119.5	O5—C16—H16A	109.5
C1—C6—H6	119.5	O5—C16—H16B	109.5
O1—C7—N1	123.95 (14)	H16A—C16—H16B	109.5
O1—C7—C1	121.23 (13)	O5—C16—H16C	109.5
N1—C7—C1	114.72 (12)	H16A—C16—H16C	109.5
N2—C8—C10	115.28 (13)	H16B—C16—H16C	109.5
N2—C8—C9	124.13 (14)		

### Hydrogen-bond geometry (Å, °)

**Table:**

*D*—H···*A*	*D*—H	H···*A*	*D*···*A*	*D*—H···*A*
N1—H1···O5^i^	0.89 (1)	2.08 (1)	2.9563 (17)	165 (2)
O5—H5···O1	0.82	1.94	2.7451 (16)	168
O4—H4···N2	0.82	1.85	2.5612 (17)	144

Symmetry codes: (i) *x*−1, *y*, *z*.
